# Okadaic Acid Is at Least as Toxic as Dinophysistoxin-1 after Repeated Administration to Mice by Gavage

**DOI:** 10.3390/toxins15100587

**Published:** 2023-09-23

**Authors:** Se Yong Park, Ju-Hee Kang, Hyun Jin Jung, Jung Ho Hwang, Hyang Sook Chun, Yeo Sung Yoon, Seung Hyun Oh

**Affiliations:** 1 Department of Anatomy and Cell Biology, College of Veterinary Medicine, Seoul National University, Seoul 08826, Republic of Korea; tpdyd2468@snu.ac.kr; 2 College of Pharmacy, Gachon University, Incheon 21963, Republic of Korea; applekjh0503@gachon.ac.kr (J.-H.K.); walkingjin86@gmail.com (H.J.J.); jhjj119@gachon.ac.kr (J.H.H.); 3 Food Toxicology Laboratory, School of Food Science and Technology, Chung-Ang University, Anseong 17546, Republic of Korea; hschun@cau.ac.kr

**Keywords:** diarrhetic shellfish poisoning, diarrhea, ascites, comparative study

## Abstract

Okadaic acid (OA) and its analogues cause diarrhetic shellfish poisoning (DSP) in humans, and risk assessments of these toxins require toxicity equivalency factors (TEFs), which represent the relative toxicities of analogues. However, no human death by DSP toxin has been reported, and its current TEF value is based on acute lethality. To properly reflect the symptoms of DSP, such as diarrhea without death, the chronic toxicity of DSP toxins at sublethal doses should be considered. In this study, we obtained acute oral LD_50_ values for OA and dinophysistoxin-1 (DTX-1) (1069 and 897 μg/kg, respectively) to set sublethal doses. Mice were treated with sublethal doses of OA and DTX-1 for 7 days. The mice lost body weight, and the disease activity index and intestinal crypt depths increased. Furthermore, these changes were more severe in OA-treated mice than in the DTX-1-treated mice. Strikingly, ascites was observed, and its severity was greater in mice treated with OA. Our findings suggest that OA is at least as toxic as DTX-1 after repeated oral administration at a low dose. This is the first study to compare repeated oral dosing of DSP toxins. Further sub-chronic and chronic studies are warranted to determine appropriate TEF values for DSP toxins.

## 1. Introduction

Phycotoxins are produced primarily by dinoflagellates and are globally distributed marine lipophilic toxins that accumulate in filter-feeding shellfishes [1]. Of the known lipophilic phycotoxins, okadaic acid (OA) and its derivatives, dinophysistoxin-1 (DTX-1), dinophysistoxin-2 (DTX-2), and dinophysistoxin-3 (DTX-3), are produced by certain species of *Prorocentrum* and *Dinophysis* genera [2]. Consumption of shellfish contaminated with OA and DTXs can lead to diarrhetic shellfish poisoning (DSP), and its typical clinical symptoms include nausea, vomiting, diarrhea, and abdominal pain [3]. Although no human death has been reported, the presence of these toxins in regularly consumed shellfish presents a public health risk and creates significant economic problems in affected areas.

OA and its derivatives inhibit the enzyme activities of protein phosphatases (PPs), especially PP2A [4], and, since PP2A is involved in a vast array of cellular processes [5,6,7,8], it has been suggested that PP inhibition is responsible for the toxic action of DSP toxins [9,10]. A rapid recombinant enzyme-based method has been developed for detecting DSP toxins based on the inhibitory effect [11] and has been used to measure the relative toxicities of OA analogues [12]. However, the PP inhibitory effect of OA and its derivatives are not sufficient to explain the diarrheagenic potencies of these toxins [13]. For example, PP inhibition by OA can both increase and reduce intestinal paracellular permeability [14,15], and increased paracellular permeability can cause fluid accumulation and diarrhea. Moreover, non-PP mechanisms involving neuropeptide Y and serotonin may also be mechanistically responsible for OA-induced diarrhea [16,17]. However, the mechanisms responsible for the toxicities of these compounds are poorly understood [13].

The Toxic Equivalency Factor (TEF) provides a measure of the toxic potency of a compound with respect to a reference compound, and it is normally used to provide a measure of the total toxicity present in foods [18]. For accurate risk assessments of toxin mixtures containing OA and its derivatives, TEF values must be properly determined for each toxin. Currently, the recommended TEF values for OA, DTX-1, and DTX-2 are 1.0, 1.0, and 0.6, respectively, but these were largely derived from acute intraperitoneal and acute oral toxicities [19,20]. In humans, DSP toxin intoxication is commonly misdiagnosed as bacterial infection and treated with antibiotics, and this situation may lead to potential inaccuracies in DSP data. In reported human DSP cases, DSP symptoms generally resolve within 2–3 days and no deaths associated with DSP have been reported. Thus, acute oral toxicities of DSP toxins that rely on the median lethal dose (LD_50_) may not be sufficiently accurate to determine TEF values or the overall toxicity of multiple DSP toxins. Furthermore, a single dose of DSP toxin would not be suitable for assessing possible toxic effects on individuals who eat shellfish frequently. Therefore, toxicity studies in vivo using repeated oral administrations at sub-lethal doses are required to establish appropriate TEF values for DSP toxins. Of the various DTXs, DTX-3 can be hydrolyzed and converted into other DSP toxins, such as OA, DTX-1, and DTX-2 [21]. Furthermore, previous studies have shown that the concentration of DTX-2 in internal organs and its oral toxicity are lower than those of OA and DTX-1 [12,22,23]. Here, we conducted a repeated oral administration toxicity study using sub-lethal doses of OA and DTX-1 to reevaluate their cumulative toxicity.

## 2. Results and Discussion

### 2.1. Acute Oral Toxicity Study of OA and DTX-1

The LD_50_ of a toxin is required to set the dose for repeated oral administration without causing death. Although many studies have provided acute oral lethality of OA and DTX-1, there are inconsistencies in the values reported. For example, doses of OA reported to cause death when administered orally vary from 400 [24] to 2000 μg/kg [25], and a single oral administration of DTX-1 at 300 μg/kg caused mouse death [26], whereas another study reported that oral administration at 750 μg/kg did not [27]. Le Hégarat et al. [28] obtained inconsistent results from two in vivo experiments. In one experiment, no mice died after oral treatment with OA at 610 µg/kg, whereas, in the other experiment, the same dose of OA killed all mice within a few hours. These inconsistencies may have been caused by differences between the mice (sex, age, or strain) or the toxins used, manufacturer, or purity. Thus, in this study, we conducted an acute oral toxicity study to determine the oral LD_50_ value of each toxin and used these to determine the sub-lethal dosage for repeated oral treatments.

**Figure 1:**
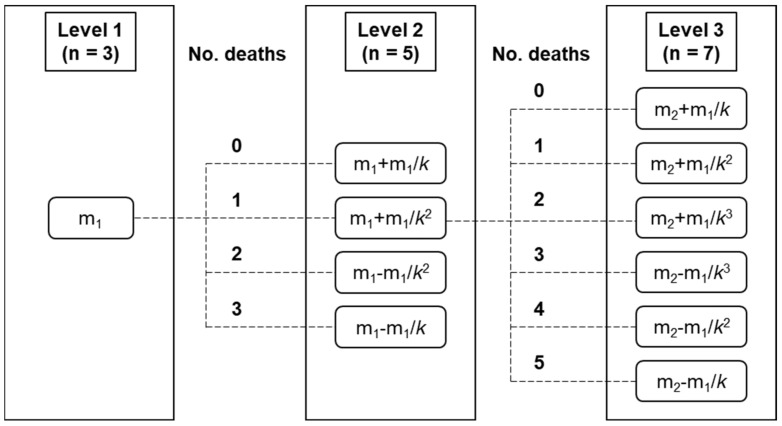
The 3-level up-and-down procedure used to determine acute oral toxicity. The 3-level up-and-down procedure is a reduced form of the 4-level Response Surface Pathway [29], and uses only 15 mice. The number of mice used at each level is shown. Doses for the next levels were based on numbers of dead mice at the previous levels using the equations written in boxes. m_1_; starting dose, m_2_; dose for level 2, *k*; adjustment factor.

The four-level Response Surface Pathway method developed by Dewi et al. [29] provides an optimal way to determine acute oral toxicity and minimize the number of mice used. Although this method required 24 mice for the 4-level up-and-down procedure, we omitted the fourth level because the author noted that 3-level is sufficient to determine the range of interest. Thus, in this study, the 3-level up-and-down procedure was performed with OA and DTX-1 (Figure 1).

The starting doses used for OA and DTX-1 were 1200 and 650 μg/kg, respectively, and the calculated adjustment factors (*k*) for OA and DTX-1 were 2.19 and 2.58, respectively. The dosages of both toxins in the second and third levels were adjusted according to the 3-level up-and-down procedure (Figure 1). The results from the three levels of the acute oral toxicity study are represented in Table 1, and the acute oral LD_50_ values of OA and DTX-1 were 1069 and 897 μg/kg, respectively.

Although the TEF values for OA and DTX-1, as recommended by EFSA [19] and the WHO [20], are 1.0 and 1.0, respectively, the in vivo acute oral toxicity study showed that the TEF value for DTX-1 is 1.5 rather than 1.0 [12,23]. Other investigators have reported that DTX-1 is more toxic than OA based on in vitro experiments [16,30,31]. In line with these results suggesting that DTX-1 is considered to be more toxic than OA, our acute oral toxicity study also showed that DTX-1 is 1.2 times more toxic than OA based on LD_50_ values. Thus, we used our LD_50_ results to set OA and DTX-1 doses for repeated oral treatments. To demonstrate dose–response relationships, three different doses of each toxin were used in the repeated dose toxicity study (Table 2).

### 2.2. Physiological Changes during the Repeated Oral Dose Study

**Figure 2:**
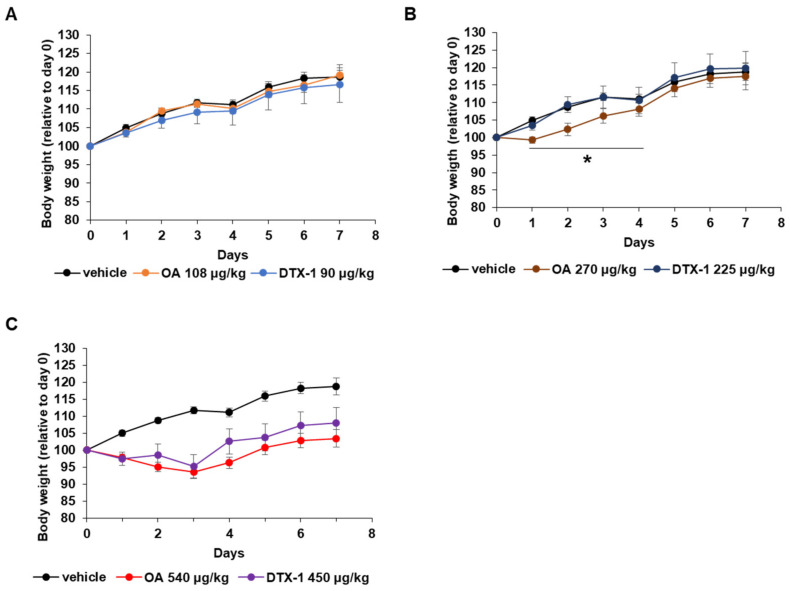
Body weight changes during the repetitive oral dosing of DSP toxins. Mouse body weights were measured daily. (**A**–**C**) Body weight changes exhibited by mice treated with low dose (**A**), moderate dose (**B**), or high dose (**C**) of OA and DTX-1 are presented as the mean ± SEM (n = 5 per group). * *p* < 0.05 versus DTX-1-treated group.

As expected, no mouse died during the repeated oral study. Since body weight loss is a common physiological parameter that reflects overall health status, mouse body weights were measured daily. The results are shown as line graphs in Figure 2. OA and DTX-1 did not affect body weights at low doses (Figure 2A), but the moderate dose of OA reduced body weights on day 1, while moderate dose of DTX-1 did not affect body weights (Figure 2B). However, this was an acute response, and body weights rebounded on day 2 and became the same as the vehicle-treated mice on day 4 (Figure 2B). When OA and DTX-1 were administered by gavage at high dose, mean body weights were significantly decreased over the first three days and then gradually recovered from day 4 to day 7, and there was no significant differences between OA and DTX-1 (Figure 2C). Although loss of body weight was temporary over the first few days, this toxic effect of OA seemed to be dose-dependent because the period of body weight loss was longer for mice treated with OA at high dose than mice treated with OA at moderate dose. Similar to our finding that loss of body weight by OA recovered after one to three days, it was previously reported that subchronically OA-treated rats exhibited temporary lethargy and body weight loss over the first few days [32].

**Figure 3:**
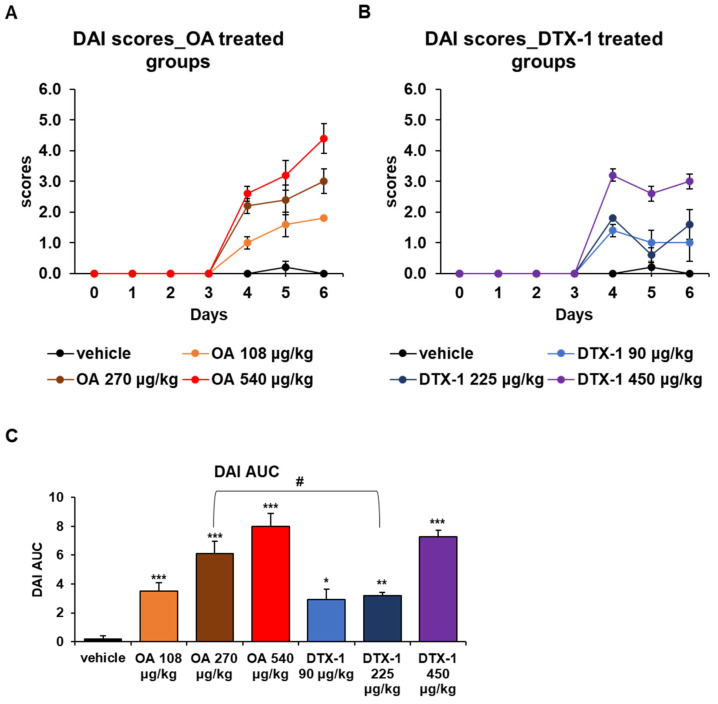
Disease activity index (DAI) score changes during the repetitive oral dosing of DSP toxins. Disease activity index (DAI) scores, the sum of stool consistency scores (0–3), and stool blood scores (0–3) were allocated daily. (**A**) DAI scores of mice treated with sublethal doses of OA are presented as the mean ± SEM (n = 5 per group). (**B**) DAI scores of mice treated with sublethal doses of DTX-1 are also presented as the mean ± SEM (n = 5 per group). (**C**) Areas under the curves (AUCs) for DAI were calculated using the graphs in (**A**,**B**). Results are presented as the mean ± SEM (n = 5 per group). Statistical significance compared to the DAI AUC of vehicle-treated mice; * *p* < 0.05, ** *p* < 0.01, and *** *p* < 0.001. # indicates *p* < 0.05.

The major health concerns about DSP in humans are gastrointestinal symptoms, such as nausea, vomiting, and diarrhea [33]. Several animal studies have also reported wet and soft feces or diarrhea after exposure to OA and DTX-1 [12,16,32]. To investigate this effect, disease activity index (DAI) scores, which include consideration of stool consistency and blood in stool, were also monitored during the experimental period. Although OA-induced diarrhea can be observed within 30 min after oral administration at 1000 μg/kg and stopped in a few hours, no evidence of diarrhea was observed during the first few days, which was probably due to the lower concentration of OA used (<1000 μg/kg). However, we observed cumulative toxic effects after repeated oral administrations. From day 4, watery stools or diarrhea were observed in OA- and DTX-1-treated groups, and DAI scores were increased dose-dependently (Figure 3A,B). In OA-treated groups, DAI scores increased significantly from day 4 to day 6, while those of DTX-1-treated groups only tended to be increased after day 4. To compare the overall diarrhetic effects of OA and DTX-1, areas under curves (AUCs) of DAI scores (DAI AUC) of both toxins were measured. DAI AUCs were significantly increased in a dose-dependent manner, and, notably, the DAI AUCs of the mice treated with the moderate dose of OA were significantly greater than those of mice treated with the moderate dose of DTX-1 (Figure 3C). The body weight loss and increased DAI scores after repeated oral treatment with OA and DTX-1 imply that the cumulative toxicity of OA might be greater than that of DTX-1.

### 2.3. Pathological Changes in the Small Intestine after Repeated Oral Doses of OA and DTX-1

**Figure 4:**
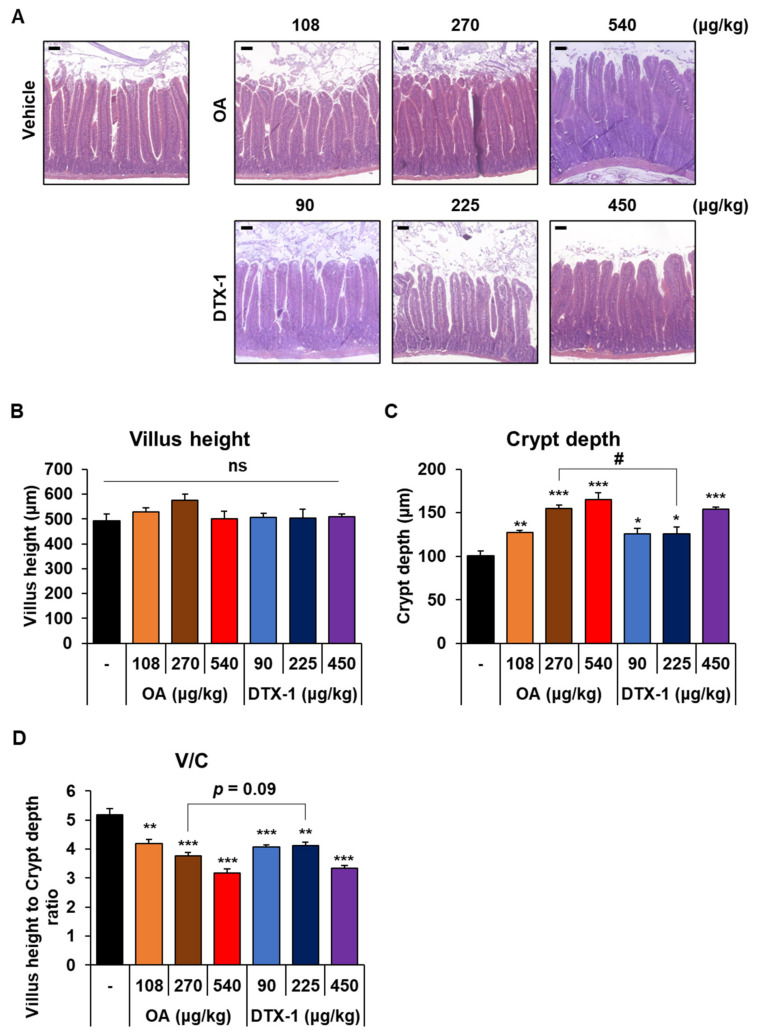
Histopathological changes in small intestine. After the experimental period, duodenums were harvested and hematoxylin and eosin (HE) stained for histopathological evaluation. (**A**) Representative images of HE-stained duodenum. Scale bar; 100 μm. (**B**,**C**) Villus height and crypt depth of duodenal villi were measured. Results are presented as the mean ± SEM. At least 7 well-oriented villi and crypts per mouse were used for measurements. (**D**) Villus height-to-crypt depth ratios were calculated. Results are presented as the mean ± SEM. Statistical significance compared to vehicle treated mice; * *p* < 0.05, ** *p* < 0.01, and *** *p* < 0.001. # indicates *p* < 0.05.

The intestinal tract is the primary target organ of OA, especially the upper part of the small intestine [24,27,34]. Therefore, to compare the intestinal toxicities of OA and DTX-1 after repeated oral administrations, we evaluated histopathological changes in duodenums isolated from mice after repetitive treatments (Figure 4A). The villus height of the duodenum was not reduced by oral administration of OA and DTX-1 repeated for 7 days (Figure 4B), which is inconsistent with previous reports that OA can induce erosion of intestinal villi [24,27,35]. However, crypt depth was significantly and dose-dependently increased by DSP toxins (Figure 4C), and this increase in crypt depth led to a significant reduction in villus-to-crypt length ratio (Figure 4D). This supports a recent suggestion that long-term, low dose OA treatment can induce cell cycle progression and colonic epithelial cell proliferation through p53 and Jak/Stat3 pathways [36]. Also, del Campo et al. [37] found OA can promote the cellular proliferation of gut cells after oral administration at a sublethal dose, and Le Hégarat et al. [28] suggested that increased mitotic figures in gut cells 24 h after gavage of OA indicate that OA increased gut cell proliferation. Since it has been known that PP2A inhibition can induce apoptosis rather than proliferation of cells, the crypt proliferation that we observed may not be due to PP2A inhibition by OA and DTX-1. Meanwhile, we treated HIEC-6 human intestinal epithelial cells with OA and DTX-1 at nanomolar concentrations. In the present study, these histopathological changes related to intestinal crypts were more severe in the duodenums of mice administered moderate dose OA than mice treated with moderate dose DTX-1 (Figure 4C,D). These results are also in line with the observed changes in body weight and DAI scores and indicate that OA might be more toxic than DTX-1 when administered repeatedly by gavage at a sublethal dose.

### 2.4. Other Toxic Effects of Repeated Oral OA and DTX-1 Administrations

Surprisingly, when abdominal cavities were opened at the necropsy, ascites was observed in mice treated with moderate or high doses OA and DTX-1. All ascites were clear or slightly cloudy and non-hemorrhagic. Ascites incidences and scores are presented in Table 3.

**Figure 5:**
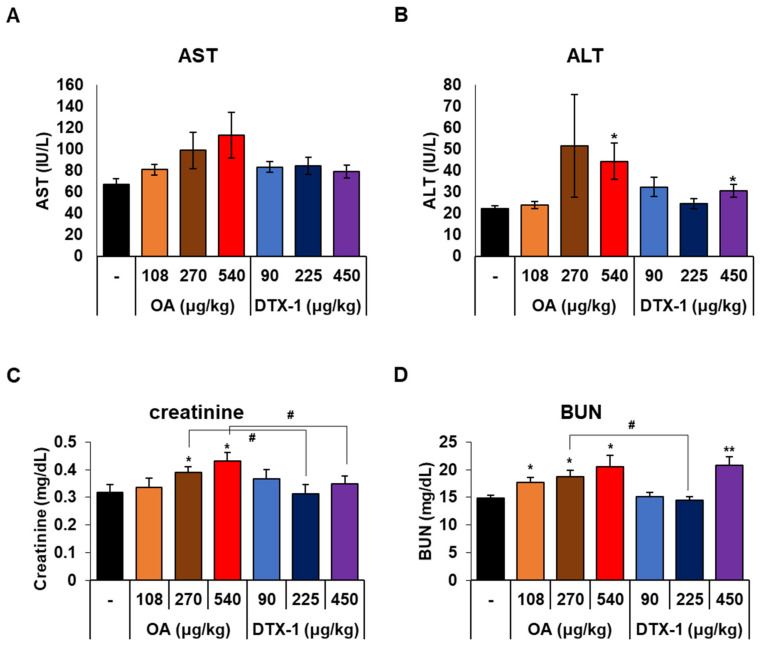
Changes in serum biomarkers of liver and kidney injury. After the treatment period, serum was collected from all mice, and serum levels of aspartate aminotransferase (AST) (**A**), alanine aminotransferase (ALT) (**B**), creatinine (**C**), and blood urea nitrogen (BUN) (**D**) were measured. Results are presented as the mean ± SEM (n = 5 per group). Statistical significance compared to vehicle treated mice; * *p* < 0.05, and ** *p* < 0.01. # indicates *p* < 0.05.

Ascites scores were determined based on visual assessment of ascites cloudiness and volume. According to the incidence and ascites scores, treatment with OA and DTX-1 dose-dependently increased ascites severity. The average ascites score of mice treated with high dose OA was significantly higher than that of mice treated with high dose DTX-1. Since ascites formation is mainly caused by chronic liver failure with cirrhosis [38], we evaluated liver injuries. Serum aspartate aminotransferase (AST) and alanine aminotransferase (ALT) (widely used biomarkers of liver injury) tended to increase dose-dependently after treatment with DSP toxins (Figure 5A,B), but no observable histopathological changes in liver tissues were observed (Figure S1A). The mean values of serum AST and ALT in mice treated with high dose OA were 113.4 and 44.4, respectively, similar to the result from Tubaro et al. [39] reporting increased AST and ALT level in mice treated with OA at 1000 μg/kg for 7 days. Interestingly, Tubaro et al. also noted that alterations in blood chemistry were less severe than those observed in their previous study [25], which involved an acute oral toxicity study following a single oral administration of OA at the same dose, suggesting that regenerative mechanisms might be induced by repeated oral administration of OA. In another study, oral administration of low dose OA for 7 days did not alter serum AST and ALT levels or liver histology [40]. It was reported more recently that a single exposure to 370 μg/kg of OA slightly increased serum AST and ALT levels but did not induce alterations in the liver [41]. In other studies, OA concentrations in the liver were low, and no pathological changes were observed [23,42]. These reports indicate that the ascites development we observed was not induced by liver damage.

Although liver cirrhosis is a major cause of ascites, renal dysfunction is a cause of non-cirrhotic ascites [43,44]. Thus, we measured serum creatinine and blood urea nitrogen (BUN) levels, which are classic biomarkers of kidney injury. In OA-treated mice, serum creatinine levels were significantly increased in a dose-dependent manner, while DTX-1 had no significant effect (Figure 5C). Similarly, serum BUN levels were dose-dependently and significantly increased after repeated administrations of OA at all doses, but only increased by high dose DTX-1 (Figure 5D). However, serum creatinine and BUN increases were insufficient to explain the development of ascites because histopathological features of renal dysfunction, such as crescents and nodular mesangial sclerosis, were not observed (Figure S1B).

Here, we report, for the first time to our knowledge, that OA and DTX-1 administration causes ascites in mice. When we expanded our literature search to include lipophilic marine biotoxins, to which OA and its derivatives belong, we found only one report of ascites induced by repeated intraperitoneal injection of azaspiracid-1 (AZA-1) into rats [45]. Clear ascites from rats injected intraperitoneally with AZA-1 was observed at 10 μg/kg and 55 μg/kg every 4 days for 15 days without liver damage and suggested that the ascites development could be evidence of the cardiotoxic activity of AZA-1. In another study, ultrastructural changes were observed in cardiomyocytes of mice treated with OA by oral administrations at 1000 μg/kg/day for 7 days [39], which suggests that the ascites we observed may have been a consequence of its cardiotoxicity.

OA can stimulate sodium secretion by intestinal cells, leading to fluid accumulation in the intestinal lumen, and this could be the mechanism of OA-induced diarrhea [46]. Moreover, fluid retention in the small intestine is another possible cause of ascites. Since intestinal crypts secrete intestinal fluids while surface epithelial cells of intestinal villi absorb fluids [47,48], the proliferation of crypts or atrophy of villi could result in fluid accumulation in intestines. In the present study, repeated oral doses of OA and DTX-1 increased crypt depth, and this may have led to fluid retention in the small intestine, which is in line with previous reports of OA-induced hypersecretion and fluid accumulation in the small intestine [39,49,50]. This abnormal fluid accumulation might cause intestinal edema [51] via increased paracellular permeability by OA and DTX-1 [52], and cause the accumulated fluid eventually be leaked from intestines to the peritoneal cavity to form ascites.

## 3. Conclusions

In the current study, first, we performed an acute oral dose toxicity study to obtain oral LD_50_ values for OA and DTX-1 in order to set appropriate sublethal doses for the repeated oral dose toxicity study. The acute oral LD50 values for OA and DTX-1 were determined as 1069 and 897 μg/kg, respectively. Based on these values, we determined the sublethal doses of OA (108, 270, or 540 μg/kg) and DTX-1 (90, 225, and 450 μg/kg). Next, we evaluated physiological and pathological changes after repeated oral dosing of OA and DTX-1 for 7 days at sublethal doses. Treatment with OA caused more severe effects, such as body weight loss, intestinal symptoms, changes in crypt depth, and severity of ascites, than DTX-1. Also, this study is the first to report the prevalence of ascites in response to administration of OA and DTX-1. According to our results, OA is at least as toxic as DTX-1 when administered orally over a long period at a sublethal dosage. Thus, to set appropriate TEF values for the regulation of DSP toxins, in vivo subchronic and chronic oral challenges with DSP toxins are warranted.

## 4. Materials and Methods

### 4.1. Diarrhetic Shellfish Poisoning Toxins

Pure okadaic acid (OA, ALX-350-003) and dinophysistoxin-1 (DTX-1, 042-33671) were purchased from Enzo Life Science (Farmingdale, NY, USA) and Fujifilm Wako Pure Chemical (Osaka, Japan), respectively. Toxins were dissolved in methanol and further diluted with PBS for oral administration.

### 4.2. Animals

Mice were maintained under a regular light/dark cycle (12 h light/12 h dark) at 22 °C and 60% humidity in a specific pathogen-free facility at Gachon University. All animal experiments were approved by the Institutional Animal Care and Usage Committee (IACUC) of Gachon University (GU1-2023-IA0014) and performed in accordance with recommended guidelines.

### 4.3. Acute Oral Dose Toxicity Study

Four-week-old male ICR mice were purchased from Orient Bio (Seongnam, Korea) and acclimated for 3 days. Only mice weighing 19 to 21 g were used. The 3-level up-and-down procedure used for the acute oral toxicity study, which was designed based on the 4-level up-and-down procedure [53] with modifications [29]. The experimental design is presented in Figure 1. At level 1, three mice were orally dosed with OA and DTX-1 at respective starting doses (m_1_) and monitored for 24 h. The starting dose (m_1_) was determined as the average of the highest (D_u_) and lowest values previously reported as oral lethal doses or LD_50_ values [24,25,42]. The adjustment factor (*k*) was calculated using Equation (1) [29].

*k* = (−m_1_ − √((4D_u_ − 3m_1_) × m_1_))/(2 × (m_1_ − D_u_)) (1)

For levels 2 and 3, five and seven mice were used, respectively, and doses at each level were determined based on numbers of dead mice at the previous level. Mice that survived more than 24 h were euthanized by CO_2_ inhalation. The median lethal dose (LD_50_) values of OA and DTX-1 were calculated using the generalized linear regression method [54].

### 4.4. Repeated Oral Dose Toxicity Study

Four-week-old male ICR mice were purchased from Orient Bio (Korea) and acclimated for 3 days. Only mice weighing 19 to 21 g were used. Thirty-five mice were randomly divided into seven groups (n = 5 per group), namely, (1) vehicle (2% (*v*/*v*) methanol in PBS), (2) OA 108 μg/kg, (3) OA 270 μg/kg, (4) OA 540 μg/kg, (5) DTX-1 90 μg/kg, (6) DTX-1 225 μg/kg, or (7) DTX-1 450 μg/kg treated groups. OA and DTX-1 doses were 10, 25, or 50% of the acute oral LD_50_ values of toxins, as determined during this study. Mice were, daily, given the corresponding vehicle, OA, or DTX-1 for 7 days by gavage. During the experimental period, body weight and disease activity index (DAI) scores were measured daily. DAI scores were calculated by summing stool consistency (0–3) and blood in stool (0–3) scores. On day 7, mice were anesthetized with isoflurane, blood was sampled by cardiac puncture, mice were euthanized by cervical dislocation, and internal organs were harvested.

### 4.5. Histological Evaluation

Formalin-fixed small intestine tissues were processed, embedded, sectioned, and stained with hematoxylin and eosin (HE). HE stained slides were scanned using Motic Slide Scanner (Motic Co., Xiamen, China) and images were captured using a Motic DSAssistant software (Motic Co., Version 2.0). Villus heights and crypt depths of small intestine were measured as previously described [55] using QuPath digital pathology software (version 0.4.1). At least 7 well-oriented villi and crypts per mouse were measured.

### 4.6. In Vitro Experiment

HIEC-6 human intestinal epithelial cells were obtained from ATCC and maintained in DMEM supplemented with 10% (*v*/*v*) fetal bovine serum and 1X penicillin/streptomycin. After being seeded on a 96-well plate, HIEC-6 cells were treated with OA and DTX-1 for 72 h, and then the cell viability was measured by MTT assay.

### 4.7. Statistical Analysis

The analysis was performed using one-way analysis of variance (ANOVA) followed by the unpaired *t*-test (two-tailed). In the case of Figure 2, the statistical analysis was performed using repeated measure ANOVA followed by pairwise t-test (Bonferroni method). All quantitative data are presented as the mean ± standard error of the mean (SEM), and *p* < 0.05 was considered statistically significant.

## Figures and Tables

**Table 1:** Acute oral toxicity study of DSP toxins based on the 3-level up-and-down procedure.

Toxin	Okadaic Acid				Dinophysistoxin-1			
Level	B.W. (g)	No. Mice	Dose (μg/kg)	Lethality (%)	B.W. (g)	No. Mice	Dose (μg/kg)	Lethality (%)
1	20.4 ± 0.2	3	1200	66.7	19.6 ± 0.1	3	650	0.0
2	20.3 ± 0.2	5	950	20.0	20.4 ± 0.3	5	902	60.0
3	20.3 ± 0.4	7	1064	57.1	20.4 ± 0.3	7	887	28.6
LD_50_ (μg/kg)	1069				897			

Mouse body weight, presented as mean ± SEMs at each level, the dose of each toxin at each level, and the lethality of each toxin at each level are shown. B.W.; body weight, LD_50_; median lethal dose.

**Table 2:** Sublethal doses of DSP toxins for repeated oral administration.

Group	Percentage of LD_50_ (%)	Okadaic Acid (μg/kg)	Dinophysistoxin-1 (μg/kg)
L; Low dose	10	108	90
M; Moderate dose	25	270	225
H; High dose	50	540	450

Sublethal doses of each toxin were determined to be 10, 25, and 50% of corresponding LD_50_ values. LD_50_; median lethal dose.

**Table 3:** Incidence and score for ascites.

	Vehicle	Okadaic Acid (μg/kg)			Dinophysistoxin-1 (μg/kg)		
		108	270	540	90	225	450
Incidence of ascites (%)	0	0	80	100	0	60	100
Ascites score	0	0	0.8 ± 0.2 **	2.2 ± 0.2 ***^,#^	0	0.6 ± 0.2 *	1.4 ± 0.2 ***

After oral administration of okadaic acid and dinophysistoxin-1 for 7 days, mice were euthanized, and abdominal cavities were opened. Ascites scores were derived based on considerations of ascites volume and cloudiness and are presented as the mean ± SEM. * *p* < 0.05, ** *p* < 0.01, *** *p* < 0.001 versus the vehicle-treated group. ^#^ *p* < 0.05 versus 450 μg/kg DTX-1-treated group.

## Data Availability

Relevant data are available from the corresponding author (S.H.O.) upon receipt of reasonable request.

## Supplementary Materials

The following supporting information can be downloaded at: https://www.mdpi.com/article/10.3390/toxins15100587/s1, Figure S1: No histopathological changes were observed in liver and kidney after repeated gavage of OA and DTX-1. Representative images of HE-stained liver (A) and Kidney (B) are shown. Scale bar; 100 μm.
