# Supplementary material for: Okadaic Acid Is at Least as Toxic as Dinophysistoxin-1 after Repeated Administration to Mice by Gavage
Source: Toxins (Basel). 2023 Sep 23;15(10):587. doi: 10.3390/toxins15100587 (PMC10611360; doi:10.3390/toxins15100587)
Supplement: Supplementary file 1 [file toxins-15-00587-s001.zip › toxins-2583811-supplementary.pdf]

toxins

Supplementary Materials

Okadaic Acid Is at Least as Toxic as Dinophysistoxin-1 after Repeated Administration to Mice by Gavage

Se Yong Park, Ju-Hee Kang, Hyun Jin Jung, Jung Ho Hwang, Hyang Sook Chun, Yeo Sung Yoon and Seung Hyun Oh

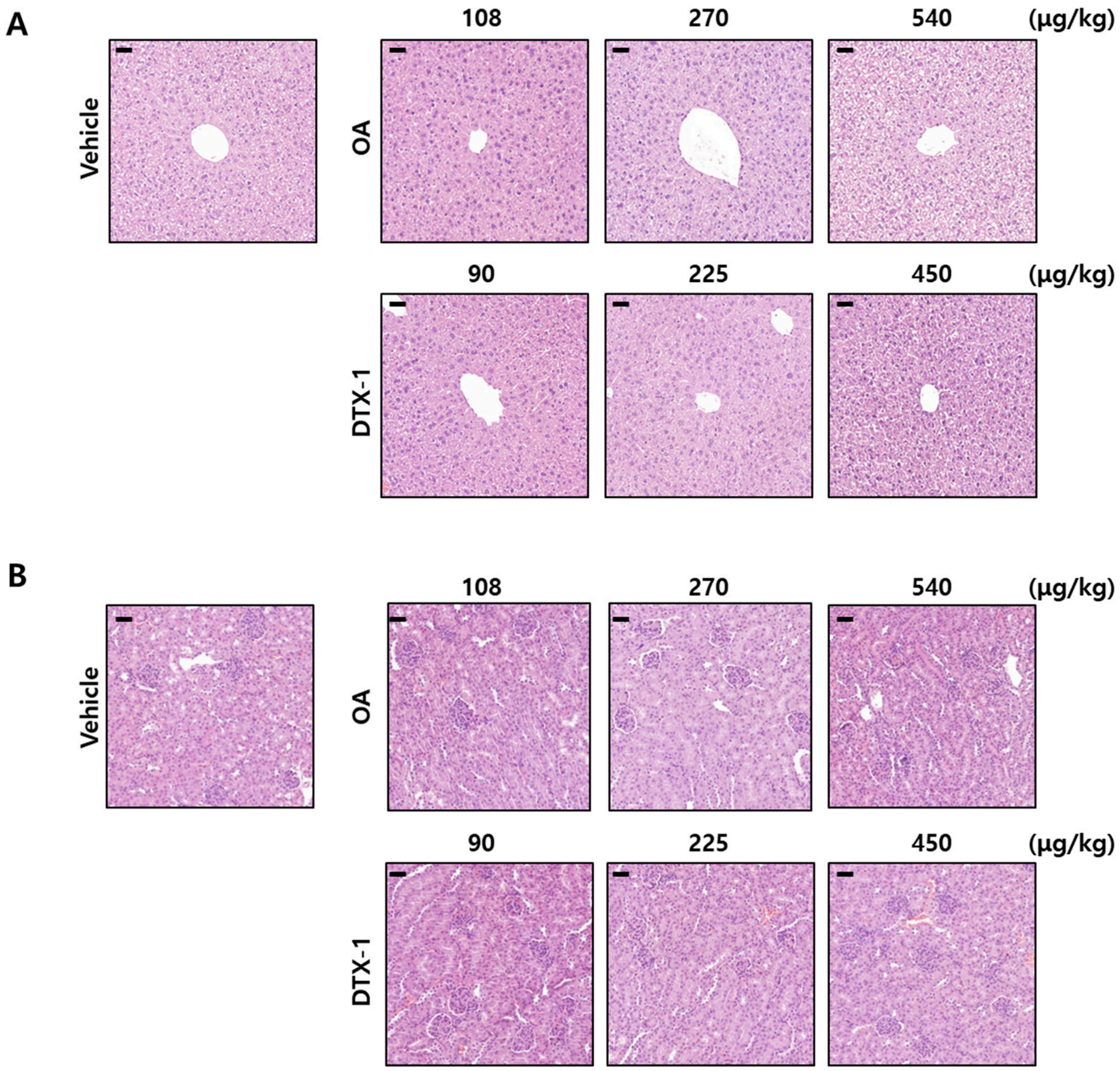

Figure S1. No histopathological changes were observed in liver and kidney after repeated gavage of OA and DTX-1. Representative images of HE-stained liver (A) and Kidney (B) are shown. Scale bar; 100 μm.
